# The amount of astrocytic GABA positively correlates with the degree of tonic inhibition in hippocampal CA1 and cerebellum

**DOI:** 10.1186/1756-6606-4-42

**Published:** 2011-11-22

**Authors:** Bo-Eun Yoon, Seonmi Jo, Junsung Woo, Jae-Hoon Lee, Taekeun Kim, Daesoo Kim, C Justin Lee

**Affiliations:** 1 WCI Center for Functional Connectomics, Korea Institute of Science and Technology (KIST), Seoul 136-791, Korea; 2 Center for Neural Science, Korea Institute of Science and Technology (KIST), Seoul 136-791, Korea; 3 Neuroscience Program, University of Science and Technology (UST), Daejeon 305-350, Korea; 4 Department of Biological Science, Korea Advanced Institute of Science and Technology (KAIST), Daejeon 305-701, Korea

## Abstract

A tonic form of synaptic inhibition occurs in discrete regions of the central nervous system and has an important role in controlling neuronal excitability. Recently, we reported that GABA present in astrocyte is the major source of tonic inhibition in cerebellum and that GABA is released through Bestrophin-1 channel by direct permeation. In this study, we screened for the presence of astrocytic GABA in various brain regions such as hippocampus, thalamus, hypothalamus and cerebellum using immunohistochemistry. We found that astrocytic GABA was present in the regions that were reported to show tonic inhibition. Because the existence of tonic inhibition in hippocampal CA1 is somewhat controversial, we compared the amount of astrocytic GABA and tonic inhibition between the hippocampal CA1 pyramidal cell layer and the cerebellar granule cell layer. Unlike cerebellar glial cells, hippocampal astrocytes did not contain GABA. The tonic inhibition was also much lower in the pyramidal neurons of hippocampal CA1 compared to the granule cells of cerebellum. Nevertheless, most of the hippocampal astrocytes expressed Bestrophin-1 channel. These data indicate that the absence of astrocytic GABA results in a low level of tonic inhibition in hippocampal CA1 region.

## Background

Tonic inhibition originates from the sustained activation of high affinity gamma-aminobutyric acid (GABA) receptors by ambient GABA [1]. Tonic current is typically seen during electrophysiological recordings as a continuous current, which is blocked by the GABA_A _receptor blockers such as GABAzine, picrotoxin and bicuculline. Because of its persistent increase in input conductance, tonic inhibition dominates over the conventional (phasic) synaptic inhibition in controlling neuronal excitability [1]. Thus, tonic inhibition plays an important role in neuronal information processing [2], and it has been implicated in epilepsy, absence seizure, sleep, memory, cognition and motor impairment [3-6].

Tonic inhibition was first identified in the cerebellum, where it is particularly prominent [7]. Recently, more studies on tonic inhibition have been performed in various regions including hippocampus and thalamus [8-11]. So far, tonic inhibition has been demonstrated in dentate granule cells [9,11], thalamocortical neurons in thalamus [5], pyramidal neurons in neocortex [12] and neurons of motor cortex [13].

Unlike those brain regions, Tonic inhibition in hippocampal CA1 region is somewhat controversial. It is reported to be absent in the pyramidal neurons of hippocampal CA1 and could be detected only in early development or in specific circumstances [10]. Other investigators reported tonic inhibition currents in pyramidal neuron after pre-incubating with GABA-transaminase inhibitor or GABA [3,14] to artificially enhance the ambient GABA level. These studies indicated that pyramidal neurons express high affinity extrasynaptic GABA receptors, ready to sense tonic GABA release. Similarly, Semyanov et al. could not observe tonic inhibition in pyramidal neurons both in stratum oriens and stratum radiatum unless the extracellular GABA concentration was elevated experimentally [15-17]. However, significant tonic inhibition was found in the interneurons of hippocampal CA1 region [16]. Therefore, the existence of tonic inhibition and source of GABA release in hippocampal CA1 region are still in question.

In cerebellum, we recently reported that Ca^2+^-activated anion channel, Bestrophin 1 (Best1), mediates tonic inhibition by releasing tonic GABA from glia. We demonstrated that GABA directly permeates through Best1 and that tonic inhibition is eliminated by silencing Best1. But the glia-specific expression of Best1 fully rescues the tonic inhibition [18]. Since the presence of both GABA and Best1 in cerebellar glial cells is critical for tonic GABA release, we predict that the same would be observed in the brain regions other than cerebellum. We previously reported that most hippocampal astrocytes express the GABA-permeable Best1 channel [19]. Therefore, we predict that the amount of astrocytic GABA positively correlates with the degree of tonic inhibition in hippocampal CA1.

To test our hypothesis, we first screened for the presence of GABA and Best1 in several brain regions and focused on astrocytic GABA and its tonic release in the hippocampal CA1 region. We show that astrocytes in the hippocampal CA1 contain negligible amount of astrocytic GABA and this correlated well with a low level of tonic inhibition currents.

## Results

### Comparison of glial GABA in hippocampus and cerebellum

To uncover the extent of astrocytic GABA, we used anti-GABA and anti-GFP antibody in GFAP-GFP transgenic mice and analyzed the percent of GABA containing cells out of all GFAP-GFP positive glial cells in several brain regions (Table 1). As opposed to GFAP marker, which only stains the cytoskeleton of astrocytes, the GFP signal from the GFAP-GFP transgenic mice is a more reliable indicator of the entire cytoplasm of astrocyte. Whereas the hippocampal CA1 region showed less than 20% GABA containing glial cells, dentate gyrus had more than 20% of astrocytic GABA. There was moderate percent of GABA containing glial cells (20~40%) in thalamus and hypothalamus. Especially, VPL (Ventral posteromedial thalamic nucleus) had more GABA containing glial cells than other sub-regions of thalamus. Consistent with our previous reports [18], we observe that most glial cells in cerebellar cortex contain GABA enough to release tonic GABA (81~100%). We excluded the cortex from the analysis because of its weak fluorescent signal in GFAP-GFP mice.

**Table 1:** GABA containing proportion of glial cells in several brain regions

Brain Region	Sub Region	GABA(+)/GFAP-GFP
Hippocampus	CA1	**+**
	Dentate gyrus	**++**
		
Thalamus	VPM (Ventral posterolateral thalamic nu)	**++**
	VPL (Vental posteromedial thalamic nu)	**++++**
	PF (Parafascicular thalamic nucleus)	**++**
	Po (Post thalamic nuclear group)	**++**
	LPLR (LP thalamic nu laterorostral)	**++**
	LPMR (LP thalamic nu, mediorostral)	**++**
		
Hypothalamus	DM (Dorsomedial hypothalamic nucleus)	**++**
		
Cerebellum	Cerebellar cortex	**+++++**

Summary table for GABA containing proportion of glial cells in several brain regions from immunohistochemistry data used anti-GABA and anti-GFP antibody in GFAP-GFP transgenic mice. The number of (+) represent percent of GABA containing cells out of all GFAP-GFP positive glial cells in each brain region. 0~20(%): + 21~40(%): ++ 41~60(%): +++ 61~80(%): ++++ 81~100(%): +++++

**Figure 1:**
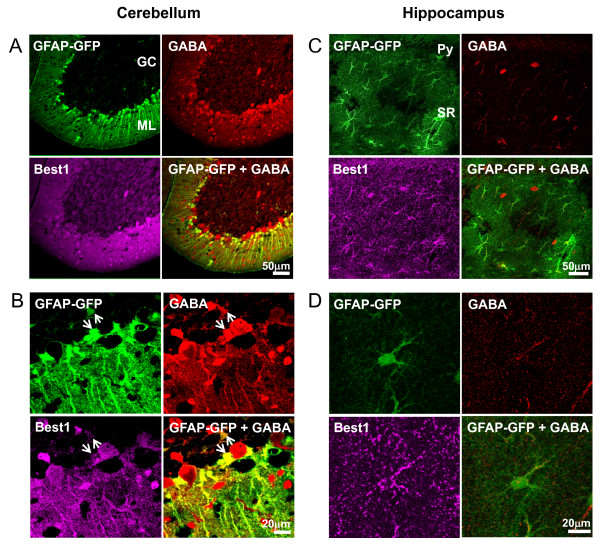
**GABA is present in cerebellar glial cells but not in hippocampal CA1 astrocytes**. Representative image of immunostaining with anti-GABA(Red), anti-GFP(Green) and anti-mBest1(Magenta) antibody showing the presence of GABA in glial cells in cerebellar cortex (A, B) and absence of GABA in hippocampal CA1 (C, D). Merge means GFAP-GFP merged with GABA. Upper panels (A, C) show lower magnification (20×, scale bar; 50 μm) and lower panels (B, C) show higher magnification (60×, scale bar; 20 μm). White arrows indicate Bergmann glial cell and lamella astrocytes in (B). GC; Granule cell layer, ML; Molecular layer, Py; Pyramidal cell layer, SR; Stratum radiatum.

Among the several brain regions we screened, we selected two regions that extremely differed in the amount of astrocytic GABA for further investigation: hippocampal CA1 and cerebellum. As we reported previously [18], most cerebellar Bergmann glial cells and lamellar astrocytes contain GABA (Figure 1A, B). To test whether hippocampal glial cells contain GABA and mediate tonic inhibition, we checked the existence of GABA and expression of Best1 in astrocytes in hippocampus using immunohistochemistry (Figure 1C, D). Interestingly, GABA was rarely shown in glial cells while the expression of Best1 was prominent in Figure 1D[19]. However, the staining pattern of glial Best1 in hippocampal CA1 was different from that in cerebellar glial cells. In cerebellum, Best1 was localized at the cell bodies and processes of Bergmann glial cell. However, in the hippocampal CA1 region, it appears to be near synapses rather than cell bodies as shown in punctate staining (Figure 1B, D).

### Comparison of tonic inhibition currents in hippocampus and cerebellum

**Figure 2:**
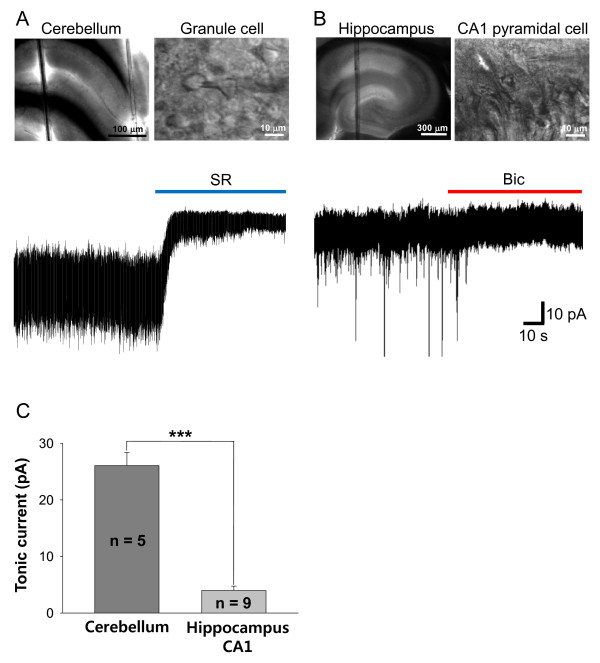
**Tonic inhibition currents recorded from cerebellar granule cells and hippocampal CA1 pyramidal cells**. A) Microphotograph images of cerebellar slice (top) and tonic current from cerebellar granule cell by GABAzine (10 μM of SR95531, bottom) B) Microphotograph images of hippocampal slice (top) and tonic current from hippocampal CA1 pyramidal cells by bicuculline (100 μM, bottom). C) Summary bar graph of tonic current from cerebellum and hippocampus. The significance of data for comparison was assessed by Student's two-tailed unpaired t-test and significance level was displayed as *** (p < 0.001).

Based on the immunohistochemical data, we expected that tonic inhibition in hippocampus should be marginal because the hippocampal glial cells contain negligible amount of GABA. To confirm this idea, we performed electrophysiological recordings from the cerebellar granule cells and the hippocampal CA1 pyramidal cells and compared the amplitude of tonic inhibition current sensitive to 10 μM SR95531 (GABAzine). From the cerebellar granule cells, we observed a considerable degree of tonic inhibition current (26.1 ± 2.3 pA, n = 5, Figure 2A, C), which was similar to our previously reported value [18]. On the other hand, the hippocampal CA1 pyramidal cells showed virtually no tonic inhibition current by 10 μM SR95531 (data not shown). It has been reported that GABA_A _receptors mediating tonic inhibition show distinct pharmacological properties based on their subunit composition [1,10]. Pyramidal neurons in hippocampal CA1 are reported to have relatively resistant tonic current against GABAzine while sensitive to picrotoxin and bicuculline [20]. Therefore, we used 100 μM bicuculline to measure the amplitude of tonic current and found that the tonic inhibition was significantly lower in hippocampal pyramidal cells (4.0 ± 0.8 pA, n = 9, Figure 2B, C) than in cerebellar granule cells.

## Discussion

We investigated the contents of astrocytic GABA in several brain regions and showed that there is a positive correlation between the amount of astrocytic GABA and the degree of tonic current. Our investigation into the amount of astrocytic GABA aptly explains the observed difference in tonic inhibition current between hippocampus and cerebellum. Furthermore, our results permit us to make predictions about other unexplored brain regions with regard to the presence of tonic inhibition.

### Correlation between amount of astrocytic GABA and tonic GABA current

We measured the amount of astrocytic GABA in various brain regions. For example, the amount of astrocytic GABA was very low and there was a minimal level of tonic inhibition (~5 pA) in hippocampal pyramidal cells in CA1. The hippocampal dentate gyrus region contained more astrocytic GABA than CA1 region, and accordingly, we could record more tonic inhibition current from the granule cell of dentate gyrus (~10 pA, data not shown). Also, the previously reported amount of thalamic tonic inhibition currents [5,8] matched our observed level of astrocytic GABA in thalamus. Especially, the cerebellum contained much astrocytic GABA and showed prominent tonic inhibition (~30 pA) [18]. With regard to tonic inhibition in the cortical regions, GFP signal was low, and we could not assess the amount of astrocytic GABA. However, the previous studies reported that there is a considerable amount of tonic inhibition in cortex such as pyramidal neurons in neocortex [12] and motor cortex [13]. Therefore, we predict that there should be a substantial amount of astrocytic GABA for tonic inhibition in the cortical regions as well. In sum, we expect that there is a strong positive correlation between astrocytic GABA and the degree of tonic inhibition throughout the central nervous system.

### Tonic inhibition in interneurons in CA1

The tonic inhibition in CA1 pyramidal cells is clearly low level and correlated well with the absence of astrocytic GABA. However, the tonic inhibition was previously reported in the interneurons of the same CA1 region [16]. An obvious question is then, how GABAergic interneurons show tonic current when there is no GABA in the surrounding astrocytes. We propose a possible explanation for this; GABA in the GABAergic interneuron can be released through Best1 channel expressed on its own membrane. GABA then self-sufficiently binds to its own high affinity GABA_A _receptor on interneuron to induce tonic inhibition. We found that the interneurons of CA1 in hippocampus also express Best1 channels using immunohistochemistry (data not shown). This possibility needs to be tested in the future experiments.

### Distinct pharmacological properties of GABA_A _receptors

We used 10 μM SR95531 (GABAzine) and 100 μM bicuculline to measure the tonic inhibition current of cerebellar granule cells and hippocampal pyramidal cells respectively. We used two different antagonists because the two cells - granule cells and pyramidal cells -have differential GABAzine sensitivity. The tonic current from the cerebellar granule cells can be measured using GABAzine because they express GABA_A _receptor that is readily blocked by GABAzine. The major subunits of tonic GABA_A _receptor of cerebellar granule cells are α6, β2, and δ [21], but the dominant subunits of pyramidal neurons are α5 and γ [3,22]. Therefore, the receptors mediating tonic inhibition show pharmacologically distinct properties on the basis of their subunit composition. Thus, pyramidal neurons in hippocampal CA1 are relatively resistant tonic current against GABAzine while sensitive to picrotoxin and bicuculline [20]. Therefore, we used bicuculline for the hippocampal CA1 pyramidal cells and GABAzine for the cerebellar granule cells.

### Function of Best1 on gliotransmission in different brain regions

We investigated not only GABA, but also the Best1 expression pattern between hippocampus and cerebellum. There was a clear distinction in the distribution of Best1 within the cell. The cerebellar glial cells expressed Best1 mostly in their cell body and processes, but the hippocampal glial cells seem to express Best1 on near synapses rather than cell body, as evidenced by the immunostaining (Figure 1D). These results suggest that Best1 function distinctively depending on the regions of brain in gliotransmission. In cerebellum, GABA is abundant in glial cells to be released tonically via Best1 to cause tonic inhibition. However, in hippocampus, GABA is not present in glial cells; rather, Best1 might release glutamate at the near synapse to affect excitatory synaptic transmission [19].

In conclusion, this investigation verified the absence of tonic inhibition in hippocampal pyramidal cells in CA1 region. Also we report the positive correlation between the amount of astrocytic GABA and the degree of tonic inhibition currents in several brain regions. Furthermore, we can expect that Best1 has different function in gliotransmission and regulation of synaptic plasticity depending on brain regions.

## Method

### Animals and housing

 C57BL/6 mice of wild-type and GFAP-GFP genotypes, of either sex, were used. All experimental procedures described below were performed in accordance with the institutional guidelines of KIST (Seoul, Korea).

### Immunohistochemistry

Adult mice were deeply anesthetized by 2% avertin (20 μl/g) and perfused with 0.1 M Phosphate buffered saline(PBS) followed by ice cold 4% paraformaldehyde (PFA). Excised brains were post-fixed overnight in 4% PFA at 4°C and immersed in 30% sucrose for 48 hrs for cryoprotection. Parasagittal cerebellar sections (30 μm) or coronal hippocampal sections were cut with a Cryostat, rinsed in PBS three times, and incubated for 1 hr with blocking solution (0.3% Triton-X, 2% normal serum in 0.1 M PBS). Sections were incubated overnight in a mixture of the following primary antibodies with blocking solution at 4°C on a shaker; rabbit anti-mouse bestrophin antibody (1:100), chicken anti-GFP antibody (1:1,000) and guinea pig anti GABA antibody (1:1,000) for GFAP-GFP mice. After washing three times in PBS, sections were incubated with corresponding secondary antibodies; conjugated Alexa 555 goat anti rabbit IgG (1:200), Alexa 488 conjugated goat anti chicken IgG (1:200), and conjugated Alexa 647 goat anti guinea pig (1:200), for one and a half hours, followed by three rinses in PBS, then mounted with fluorescent mounting medium. A series of fluorescence images were obtained with an Olympus confocal microscope (FV1000) and images were processed for later analysis using FLUOVIEW software.

### Slice recording

Animals were deeply anesthetized with halothane. After decapitation, the brain was quickly excised from the skull and submerged in ice-cold cutting solution that contained (in mM): 250 Sucrose, 26 NaHCO3, 10 D(+)-Glucose, 4 MgCl2, 3 myo-inositol, 2.5 KCl, 2 Sodium pyruvate, 1.25 NaH2PO4, 0.5 Ascorbic acid 0.1 CaCl2, and 1 Kynurenic acid, pH 7.4. All solution was gassed with 95% O2-5% CO2. After trimming either side of the vermis, 250 μm thick parasagittal slices were cut using a microtome and transferred to extracellular ACSF solution: 126 NaCl, 24 NaHCO3, 1 NaH2PO4, 2.5 KCl, 2.5 CaCl2, 2 MgCl2, and 10 D(+)-Glucose, pH 7.4. For hippocampus, 300 μm thick coronal slices were used. Slices were incubated at room temperature for at least one hour prior to recording. Slices were transferred to a recording chamber that was continuously perfused with ASCF solution (flow rate = 2 ml/min). Slice chamber was mounted on the stage of an upright Olympus microscope and viewed with a 60X water immersion objective (NA = 0.90) with infrared differential interference contrast optics. Cellular morphology was visualized by CCD camera and Axon Imaging Workbench software. Whole-cell recordings were made from granule cell somata located in lobules 2-5 or pyramidal cells located in hippocampal CA1 region. The holding potential was -70 mV. Pipette resistance was typically 10-12 MΩ for granule cells and 5-7 MΩ for pyramidal cells and pipette was filled with an internal solution: 135 CsCl, 4 NaCl, 0.5 CaCl2, 10 HEPES, 5 EGTA, 2 Mg-ATP, 0.5 Na2-GTP, 10 QX-314, pH adjusted to 7.2 with CsOH (278-285 mOsmol). Electrical signals were digitized and sampled at 50 μs intervals with Digidata 1440A and Multiclamp 700B amplifier (Molecular Devices) using pCLAMP 10.2 sofware. Data were filtered at 2 kHz.

### Data analysis and statistical analysis

Off-line analysis was carried out using Clampfit, Minianalysis, SigmaPlot and Excel software. Numerial data are presented as means ± S.E.M. The significance of data for comparison was assessed by Student's two-tailed unpaired t-test and significance level was displayed as * (p < 0.05), ** (p < 0.01), ***(p <0.001).

## Competing interests

The authors declare that they have no competing interests.

## Authors' contributions

BEY carried out electrophysiology, immunohistochemistry and wrote the manuscript. SJ performed electrophysiology and immunohistochemistry. JW carried out electrophysiology. JHL and TK performed immunohistochemistry. CJL designed the most of experiments, edited the manuscript, and coordinated entire project. All authors read and approved the final manuscript.
